# Good Rules for Dyspeptics

**Published:** 1890-05

**Authors:** 


### SOME GOOD RULES FOR DYSPEPTICS.

We published in our March number a quite lengthy article on the above subject, but some of our dyspeptic patrons complained that its prohibited list was too sweeping and left them a too scanty regimen. We take the following simple rules from the *Phrenological Journal*, which, if followed out, will do much towards relieving the distress occasioned by this quite prevalent malady.

1. Eat two meals a day.

2. Eat slowly, masticate the food very thoroughly, even more so, if possible, than is required in health.

3. Avoid drinking at meals; at most take a few sips of warm, unstimulating drink at the close of the meal, if the food is very dry in character.

4. In general dyspeptic stomachs manage dry food better than that containing much fluid ; so avoid light soups.

5. Eat neither very hot nor cold food. The best temperature is about that of the body. Avoid exposure to cold soon after eating.

6. Be careful to avoid excess in eating. Eat no more than the wants of the system require. Strength depends not on what is eaten, but on what is digested.

7. Never take violent exercise of any sort, either mental or physical, just before or just after a meal. It is not good to sleep immediately after eating.

8. If it is thought necessary to eat three times a day make the last meal very light. For most dyspepties two meals are better than more.

9. Never eat a morsel of any sort between meals.

10. Never eat when very tired, whether exhausted from mental or physical labor.

11. Never eat when the mind is worried or the temper is ruffled, if it is possible to avoid doing so.

12. Eat only food that is easy of digestion, avoiding complicated and indigestible dishes, and take but two or three kinds at a meal.

13. Most persons will be benefited by the use of oatmeal, wheat meal, or graham flour, cracked wheat, and other whole grain preparations, though many will find it necessary to avoid vegetables, especially when fruits are taken.

14. Some kind of fruit, ripe, fresh or in the simple form of stewed or canned, should be eaten at breakfast, as fruit promotes digestion. The use of fruit obviates the necessity of drinking while eating, and for those who have been habituated to drinking, a dish of stewed apples or prunes will serve as well.

